# Weakly Hydrated Solute of Mixed Hydrophobic–Hydrophilic Nature

**DOI:** 10.1021/acs.jpcb.4c02429

**Published:** 2024-06-24

**Authors:** Aneta Panuszko, Maciej Śmiechowski, Maciej Pieloszczyk, Adrian Malinowski, Janusz Stangret

**Affiliations:** Department of Physical Chemistry, Faculty of Chemistry, Gdańsk University of Technology, Narutowicza 11/12, 80-233 Gdańsk, Poland

## Abstract

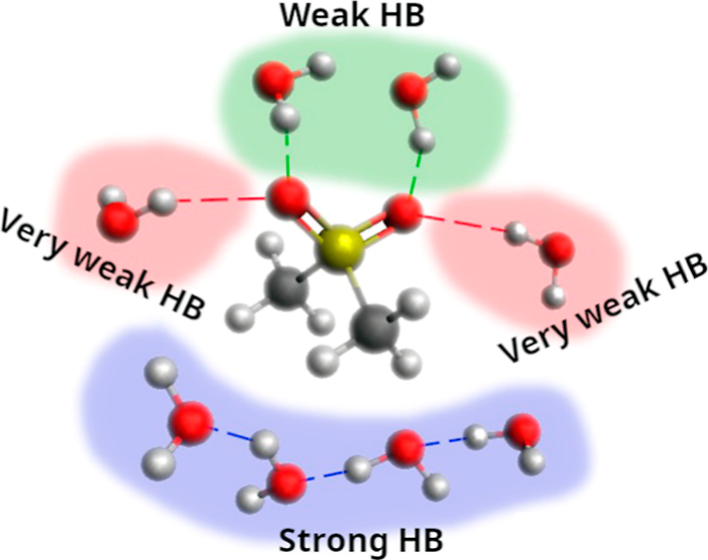

Infrared (IR) spectroscopy is a commonly used and invaluable tool in studies of solvation phenomena in aqueous solutions. Concurrently, density functional theory calculations and ab initio molecular dynamics simulations deliver the solvation shell picture at the molecular detail level. The mentioned techniques allowed us to gain insights into the structure and energy of the hydrogen bonding network of water molecules around methylsulfonylmethane (MSM). In the hydration sphere of MSM, there are two types of populations of water molecules: a significant share of water molecules weakly bonded to the sulfone group and a smaller share of water molecules strongly bonded to each other around the methyl groups of MSM. The very weak hydrogen bond of water molecules with the hydrophilic group causes the extended network of water hydrogen bonds to be not “anchored” on the sulfone group, and consequently, the MSM hydration shell is labile.

## Introduction

Liquid water is known as the “matrix of life” as it constitutes the environment for all cellular life on Earth and actively shapes the stability, structure, dynamics, and biological function of proteins and other biomolecules.^1−3^ The intracellular water provides a hydration environment to both hydrophobic and hydrophilic exposed fragments of biopolymers, which are frequently interspersed on the solvent accessible surface of these molecules.^4−7^ However, this solvent is never pure water, but rather a complex solution of ionic and nonionic solutes that heavily modify its structure and dynamics and that themselves interact with biomolecules in a multitude of ways.^8,9^

The classical picture of the hydrophobic hydration, based on purely entropic considerations and harking back to the “iceberg formation” hypothesis formulated by Frank and Evans,^10^ posits an ordering of water structure around hydrophobic solutes as in ice. This would imply measurable changes in the infrared (IR) spectrum of water, which is notably different for liquid water and ice I_h_ in the OH stretching range.^11^ The experimental confirmation of this conclusion had been elusive for decades, but has been recently obtained by Grdadolnik et al. using high-pressure IR spectroscopy of aqueous solutions of small purely hydrophobic gases.^12^ However, from a structural point of view the hydration shell of aqueous krypton is more loosely defined then in the respective clathrate hydrate.^13^ The hydration shell of alkanes is also not clathrate-like, but rather dynamic and driven by van der Waals (vdW) attractive forces.^14^

On the other hand, opinions on the water structure near the hydrophobic fragments of amphiphilic solutes have varied considerably (see, e.g., ref (15) for a review). Solutes which possess both hydrophilic and hydrophobic fragments still await a thorough explanation of their complex hydration.^4,16^ It is known that the charged form of the polar fragment of the solute severely influences the hydrophobic hydration of the alkyl side chains, more than the corresponding neutral form.^17,18^ Weakly hydrated groups, such as the SO_3_^–^ in *N*,*N*,*N*-trimethyltaurine,^19^ are now known to severely disrupt the hydrophobic hydration shell of nearby alkyl groups.

Recently, we studied the hydration of dimethyl sulfoxide (DMSO) finding that the rather strong hydration of the S=O group favors the emergence of a clathrate-like cage around the entire solute.^20^ Here, we examine the hydration of the oxidized sulfonyl analogue of DMSO, namely, methylsulfonylmethane (MSM). Known also as dimethyl sulfone (DMSO_2_), MSM occurs naturally in mammalian metabolism as one of the major end products in the methionine degradation pathway via DMSO.^21^ It is currently gaining popularity as a relatively nontoxic dietary supplement.^22,23^ Unlike DMSO, its applications as a solvent are limited by its high melting point (382 K),^24^ but it is being investigated as a promising high temperature solvent for the electrodeposition of aluminum^25^ and as a component of deep eutectic solvents for application in aqueous lithium-ion batteries.^26^ For the present purpose, MSM displays a weakly hydrated sulfonyl group directly connected to methyl groups, thus providing a convenient framework to test the hypothesis that the presence of weakly hydrated uncharged polar groups can severely disrupt the hydrophobic hydration shell of neighboring alkyl substituents.

Following our previous investigations,^20,27,28^ we leverage the versatility and sensitivity of Fourier transform infrared (FTIR) spectroscopy as an ideal tool to study the solute influence on the solvation environment, particularly in aqueous systems. The OD stretching vibration of semiheavy water (HDO) served as a probe for the structural and energetic state of the solvent. The spectra of HDO exhibit a notable absence of experimental and interpretative challenges commonly associated with spectra of H_2_O. The experimental findings were supported and interpreted through the utilization of density functional theory (DFT) calculations. The accurate solvation shell picture and strong interpretative power of the computational IR spectra were provided by ab initio molecular dynamics (AIMD) simulations. Thus, we have delivered a comprehensive and coherent perspective on the hydration of MSM and its far reaching implications for other weakly hydrated solutes.

## Materials and Methods

### Chemicals and Solutions

Methylsulfonylmethane (99%, Alfa Aesar) was used as supplied. A weighted amount of MSM was dissolved in deionized water (κ < 0.01 μS·cm^–1^) in order to prepare a stock solution with a molality *m* ≈ 0.975 mol·kg^–1^. Further solutions with progressively decreasing molality were obtained by diluting the stock solution with an appropriate amount of deionized water. Each of the solutions was subsequently divided into two parts. Sample solutions for the HDO spectra were made by adding D_2_O (isotopic purity 99.96%, Aldrich) to one part of the solution in an amount of 4 wt % relative to H_2_O (H_2_O + D_2_O ⇄ 2HDO, *K* ≈ 4)^29^ contained in the solution, while reference solutions (without D_2_O) were prepared by adding H_2_O to the other part of the solution in molar equivalent to the D_2_O addition in the sample solution. As a result, we obtained a series of MSM solutions (references and samples) with molalities of about 1.0, 0.8, 0.6, 0.4, 0.2, and 0.1 mol·kg^–1^.

All solutions were prepared by weight, and their densities were determined with the Anton Paar DMA 5000 densitometer at 25.000 ± 0.001 °C.

### FTIR Spectroscopy

FTIR spectra of aqueous solutions of MSM were recorded with a Nicolet 8700 spectrometer (Thermo Scientific) controlled by OMNIC 7.2 acquisition software (Thermo Electron Corporation). The spectra were averaged over 128 independent scans registered with the resolution of 4 cm^–1^ in the 500–5000 cm^–1^ spectral range. The spectrometer was purged with dry nitrogen during the measurements. A liquid transmission cell (model A145, Bruker Optics) was equipped with two CaF_2_ windows separated by poly(tetrafluoroethylene) spacers. The cell’s path length was 28.44 μm, as determined interferometrically. The temperature of the cell was kept constant at 25.0 ± 0.1 °C by means of a circulating temperature controller (Julabo F12) and monitored by using an electronic thermometer with a thermocouple placed inside the cell.

### Analysis of FTIR Spectra

The spectra were analyzed using commercial software: GRAMS/AI version 9.3 (Thermo Fisher Scientific Inc., Waltham, MA, USA) and RazorTools/8 (Spectrum Square Associates, Inc., Ithaca, NY, USA) are run under GRAMS/AI.

The difference spectral method was applied to extract the MSM-affected HDO spectrum, which was extrapolated to the very diluted solution limit on the basis of the spectral series measured for different molalities of the aqueous solutions. The main assumption of the method is that the water in solution can be divided into two additive contributions: bulk water (spectrally identical to pure water) and solute-affected water (modified by interactions with the solute). The latter is understood in terms of the entire influence of the solute, although it can be, in principle, separated later into contributions of different functional groups of the solute, as demonstrated below.

The spectrum of solute-affected water in the high-dilution approximation can be obtained using the following eq 1
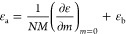
1where ε_a_ and ε_b_ are, accordingly, the molar absorption coefficients of affected water and bulk water at each wavenumber value, *N* is the number of moles of water affected by 1 mol of solute (affected number), *M* is the mean molar mass of water (taking into account the mass of D_2_O in HDO spectra) (kg·mol^–1^), and *m* is the molality of the solute (mol·kg^–1^). The derivative  of the molar absorption coefficient value versus molality for *m* = 0, at each wavenumber, is obtained by approximating the ε versus *m* dependence with an appropriate polynomial fitting function.

The spectral band shapes can be transformed to the interatomic oxygen–oxygen distance distribution functions, *P*(*R*_OO_), using an empirical function^30^
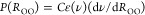
2where *C* is a normalization constant and (dν/d*R*_OO_) is obtained by analytically differentiating the relation from eq 3
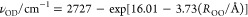
3

This relationship is based on the position of HDO bands in the solid hydrates, which was measured with the use of infrared spectroscopy, and the respective intermolecular distances determined by diffraction methods.^30^

Details concerning the extraction, interpretation of the solute-affected water spectrum, as well as the transformation of HDO spectral contours into the probability distribution of the intermolecular oxygen–oxygen distance, *P*(*R*_OO_), have been described in detail in refs (31−33) and also in the Supporting Information of refs (34 and 35).

### DFT Calculations

Structure of MSM and structures of its complexes with water molecules were optimized using the density functional theory (DFT) level with the B3LYP hybrid exchange–correlation functional^36,37^ and 6-311++G(d,p) basis set.^38^ The conductor-like polarizable continuum model (CPCM) of the self-consistent reaction field theory was used to model the solvent environment.^39,40^ The D3 version of Grimme’s empirical dispersion correction, including the Becke-Johnson damping (GD3-BJ), was applied.^41^ The analysis of resulting wave function files, involving the reduced density gradient (RDG) method,^42^ was performed with the Multiwfn v.3.3.9 software.^43^ The RDG method allowed visualization of weak noncovalent interactions.

The optimization was performed first in the gas phase and then using the CPCM model. We gradually added water molecules to the optimized MSM structure, and hydrated complexes were optimized. We carried out the calculations until a closed ring of water molecules surrounding the MSM methyl groups was formed. The calculated structures exhibited no negative vibration frequencies; thus, they corresponded to the local energy minima.

Gutmann’s donor numbers (DNs) were calculated with the method proposed by Smiatek and Miranda-Quintana.^44^ DN values therein are fitted to values of solvation energies obtained according to the conceptual density functional theory. Necessary highest occupied molecular orbital and lowest unoccupied molecular orbital energies of MSM were calculated in the gas phase using DFT with B3LYP functional^36,37^ and def2-TZVP basis set^45^ with GD3-BJ dispersion correction.^41^ The original Python code was modified to allow for the calculation of unknown DNs from results of the curve fit. The original data set was used, apart from DMSO parameters, which were used for calculation of the DMSO DN as if it were unknown, in order to compare the results with the calculated MSM DN.

All calculations were performed using the Gaussian 09 rev.D01 software^46^ and the ORCA 5.0.4 program system.^47,48^ The program Avogadro v.1.2.0^49^ was used for the preparation of input data and visualization of computed results.

### AIMD Simulations

AIMD simulations were performed with the DFT-based Quickstep electronic structure module implemented in the CP2K 6.0 computational suite.^50,51^ We applied the BLYP functional^36,37^ together with the DFT-D3 empirical dispersion correction with zero damping.^41^ The cutoff for the latter was set to 16 Å. Quickstep implements a mixed Gaussian type atomic orbitals (AOs) plus plane waves (PWs) basis set scheme (known as GPW),^52^ and we used a TZV2P AO basis set combined with a 500 Ry cutoff for the PW expansion of the electron density. Valence electrons were treated explicitly, while core electrons were represented by GTH pseudo potentials.^53^

We studied the MSM(H_2_O)_80_ system (*m* ≈ 0.624 mol·kg^–1^), contained in a cubic supercell with periodic boundary conditions applied (*L* ≈ 13.63 Å). In order to compare directly to the FTIR spectra of HDO/H_2_O, all water hydrogen atoms were given the mass of deuterium, i.e., we effectively simulate our solute in D_2_O. The initial volume of the system was chosen to reflect the experimental density of heavy water^54^ combined with the apparent molar volume of MSM at the simulated concentration.^55^ The simulations of pure D_2_O employing the same protocol were published previously.^20^

The system was initially equilibrated for 20 ps with a time step of 0.5 fs in the *NVT* ensemble using massive Nosé–Hoover chain thermostatting.^56^ In order to meaningfully compare with the experimental data measured at 298.15 K, we use the common approach of temperature overscaling in order to avoid the slow dynamics regime, common for generalized gradient approximation-based functionals.^57^ The extent of this scaling is slight for D_2_O and the target temperature is set to 323.15 K.^58^ After the equilibration period, 20 initial conditions were sampled every 3 ps from a further *NVT* simulation to initialize microcanonical (*NVE*) trajectories of 20 ps length each. During these runs, the centers of maximally localized Wannier functions (MLWFs)^59^ were determined every 2 fs. All analyzed observables were averaged over the 20 *NVE* trajectories yielding proper canonical averages.

Instantaneous molecular dipole moments were obtained classically by summing over positive nuclei and negative MLWF centers. IR spectra were calculated as Fourier transforms of time correlation functions of dipole moment finite differences^60,61^ using dipolar decomposition schemes for solute–solvent systems introduced previously by us, see refs (61 and 62) for details. The spectral resolution was set to 1 cm^–1^ by setting the upper limit of the correlation time to ∼16.67 ps and the obtained spectra were smoothed by passing through a 20 cm^–1^ Gaussian filter. Numerical Kramers–Kronig transform^61^ was used to remove the refractive index contribution to the IR spectra using the experimental optical frequency refractive index of D_2_O, *n*_D_ = 1.328.^63^

## Results

### Experimental FTIR Spectra of MSM in Aqueous Solutions

**Figure 1:**
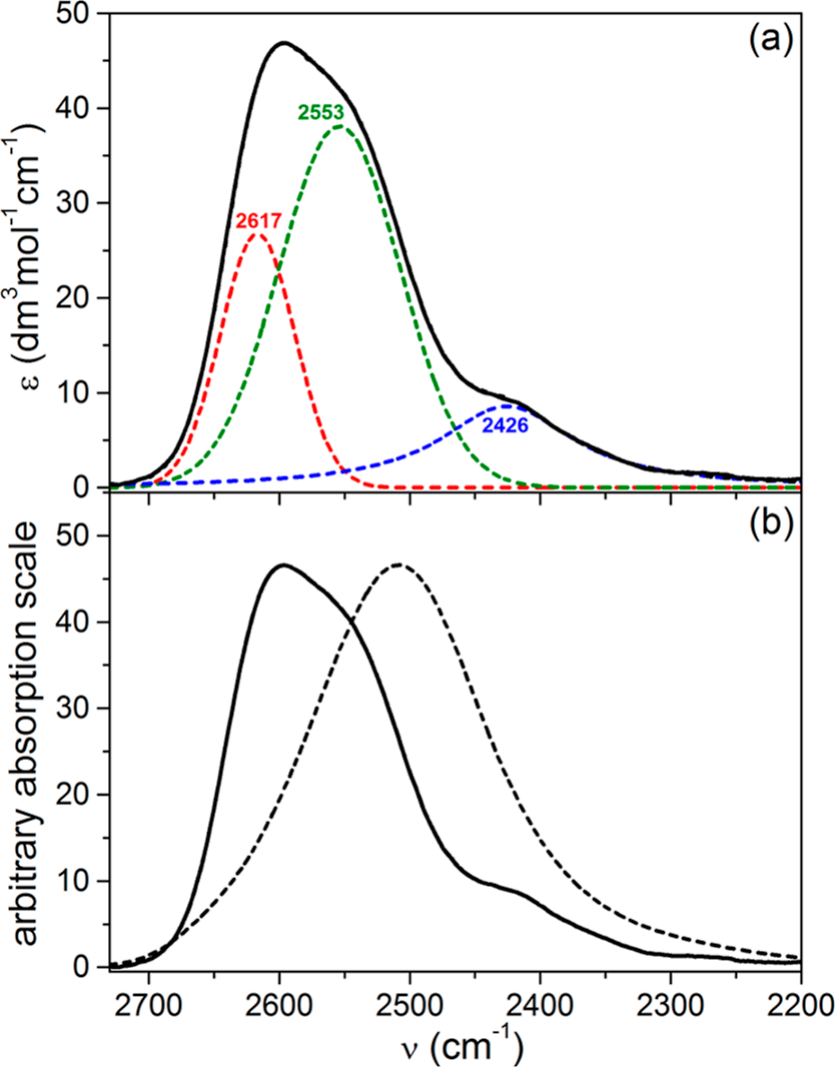
(a) Decomposition of HDO spectrum affected by MSM (in the OD stretching region) into component bands (with values corresponding to the position of the maximum of the band). Solid line: original affected spectrum; dotted line: sum of the component bands (covered by the solid line of the original spectrum); and dashed lines: OD component bands. (b) MSM-affected HDO spectrum in the OD stretching region (solid line) and the bulk HDO spectrum (dashed line). The spectra have been scaled to the same maximum absorption value for better comparison.

**Figure 2:**
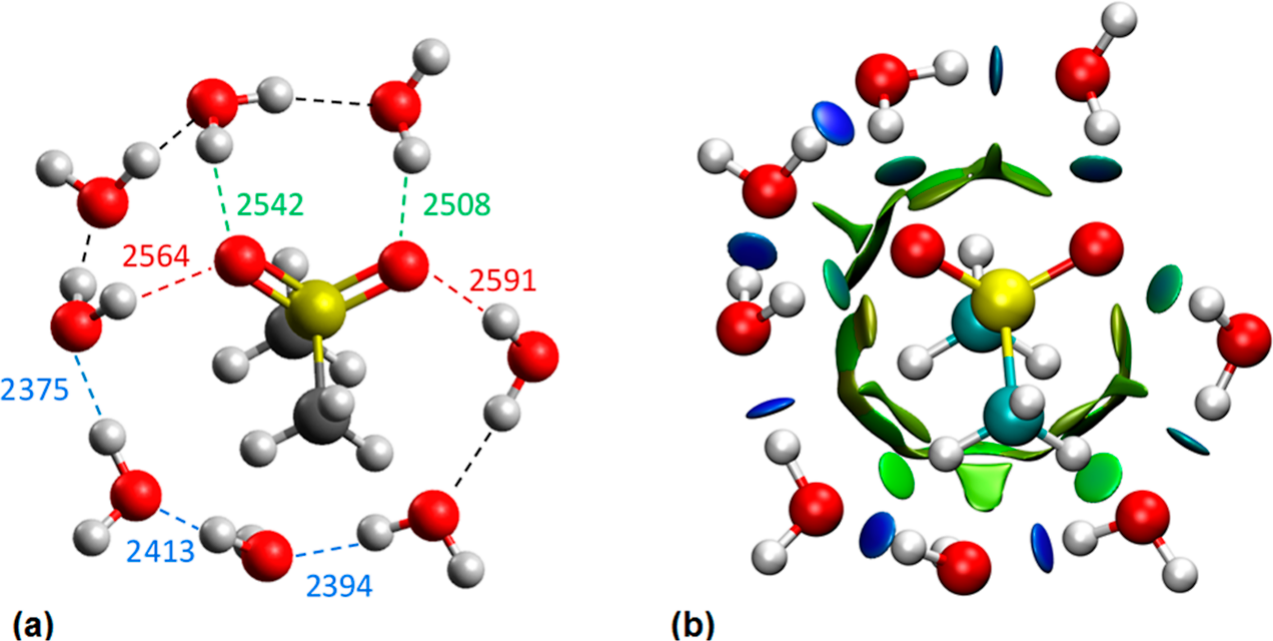
(a) Optimized structure of the hydrated MSM complex calculated in the CPCM model at the B3LYP/6-311G++(d,p) level of theory and corresponding vibrational frequencies (cm^–1^) obtained from transformation of interatomic oxygen–oxygen distances (*R*_OO_) to the OD band position of HDO (ν_OD_) with the aid of the empirical relation (eq 3). Hydrogen bonds are marked with dashed lines. The colors of hydrogen bonds together with the vibration frequencies correspond to the colors of the component bands shown in Figure 1a. (b) Visualization of weak interactions analysis by the RDG method for MSM complex with water molecules (from Figure 2a). Blue or green disks denote well-focused hydrogen bonds (light blue/green–weak HB, dark blue–strong HB), green/olive patches indicate weak van der Waals interactions.

In the spectrum of MSM-affected water (Figure 1a), we can distinguish three main component OD bands with corresponding band positions. In order to facilitate the interpretation of these component bands, we obtained an optimized structure of the hydrated MSM complex (Figure 2a) by using DFT calculations within the CPCM model of water as a solvent. The intermolecular oxygen–oxygen distances (*R*_OO_) from this structure were converted to vibrational frequencies of the OD bands (ν_OD_) utilizing an empirical relation (eq 3).

The band located at high wavenumbers (2617 cm^–1^) corresponds to the interactions of water molecules with the oxygen atoms of the sulfone group, which simultaneously interact with the water molecules around the MSM hydrophobic groups (see Figure 2a). The average frequency of vibrations from the MSM hydration structure (Figure 2a, frequencies marked in red) corresponding to these interactions is 2578 ± 14 cm^–1^. Component bands located at 2553 cm^–1^ characterize water molecules involved in hydrogen bonding with oxygen atoms of the sulfonyl group and interacting with each other. The average frequency of these vibrations from the MSM hydration structure (Figure 2a, frequencies marked in green) is 2525 ± 17 cm^–1^. Accordingly, the interactions of the water molecules with the sulfone group are very weak, which is also evidenced by the light blue disks in Figure 2b. On the other hand, the smallest component band at the low-wavenumbers position (2426 cm^–1^) corresponds to the interactions between water molecules around the methyl groups of the MSM molecule, supported by simultaneous interactions with methyl hydrogens (light green disks in Figure 2b).

The average vibration frequency from the MSM hydration structure (Figure 2a, frequencies marked in blue) is 2394 ± 19 cm^–1^. This strong interaction between water molecules around the hydrophobic groups of the MSM is visible in Figure 2b as dark blue discs.

The “affected” spectrum provides very important information about the energetic and structural properties of water molecules in the hydration sphere of methylsulfonylmethane. The spectral contours of water affected by MSM and pure water, scaled to the same height, are shown in Figure 1b.

**Figure 3:**
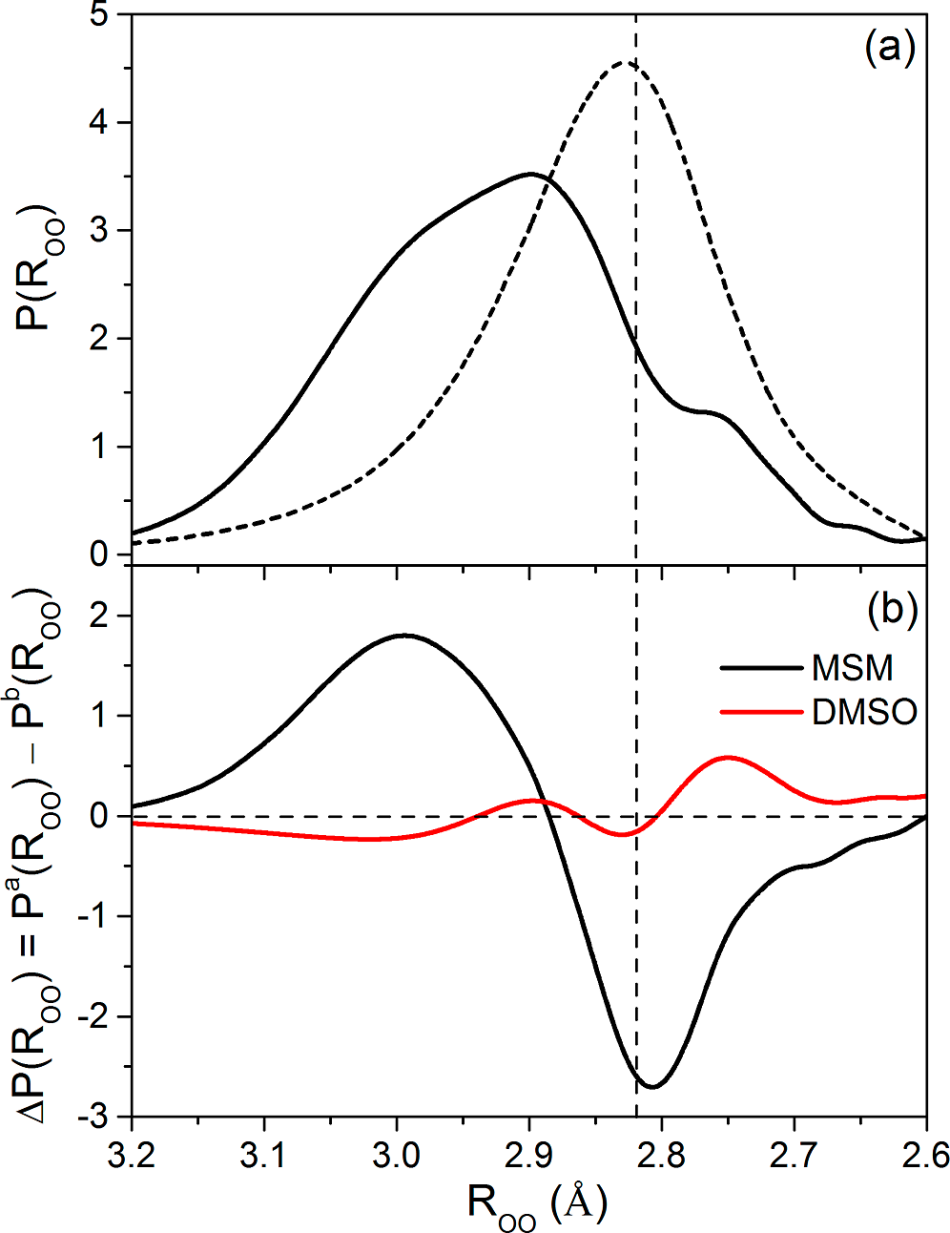
(a) Interatomic oxygen–oxygen distance distribution function derived from the HDO spectrum affected by MSM (solid line) and bulk water spectrum (dashed line) (Figure 1b) (b) Differences between interatomic oxygen–oxygen distance distribution function of solute-affected water, *P*^a^(*R*_OO_) and bulk water, and *P*^b^(*R*_OO_) for MSM and DMSO (from ref (20)). The vertical dashed line corresponds to the value of the most probable oxygen–oxygen distance for bulk water (2.826 Å, see Table 1).

These spectra were converted into the interatomic oxygen–oxygen distance distribution functions, *P*(*R*_OO_), using (eq 2). The results of these transformations are shown in Figure 3a, with the difference in probability distributions for MSM-affected and bulk water additionally shown in Figure 3b. The *P*(*R*_OO_) distribution obtained for MSM-affected water takes into account water molecules involved in hydrogen bonds with other water molecules and oxygen atoms of the sulfone group. The main parameters of the spectra of MSM-affected water and bulk water, together with the intermolecular oxygen–oxygen distances, *R*_OO_, are summarized in Table 1.

**Table 1:** Parameters of HDO Bands of Water Affected by MSM, and Bulk Water (Figure 1B), and the Respective Intermolecular Oxygen–Oxygen Distances^a^

solute	*N*^b^	ν^o^_OD_^c^	ν_OD_^m^^d^	fwhh^e^	*I*^f^	*R^o^*_OO_^g^	*R*_OO_^m^^h^
bulk water		2509 ± 2	2496 ± 2	162 ± 4	9986	2.826 ± 0.003	2.844 ± 0.003
MSM	3.2 ± 0.5	2596 ± 2	2547 ± 2	146 ± 4	7619	2.897 ± 0.003	2.925 ± 0.003

a *R*_OO_ errors have been estimated on the basis of the HDO band position errors.

b Affected number, equal to the number of moles of water affected by 1 mol of solute.

c Band position at maximum (cm^–1^).

d Band position at center of mass (cm^–1^).

e Full width at half-height (cm^–1^).

f Integrated intensity (dm^3^·mol^–1^·cm^–1^).

g The most probable O···O distance (Å).

h Mean O···O distance (Å).

The shift of the center of mass band value, ν_OD_^m^ (related to the mean hydrogen bond energy of water molecules), toward higher value with respect to the ones corresponding to pure water (Table 1), indicates that water–water hydrogen bonds are on average weaker in the surrounding of MSM. This conclusion is also supported by the longer oxygen–oxygen distance between water molecules in “affected” water compared to pure water. Based on the above observations, it can be concluded that MSM should be classified as a water “structure-breaking” solute.

### Hydration Shells and Infrared Spectra of Aqueous MSM from AIMD Simulations

While, in principle, force field-based simulations can provide a much larger time scale of the trajectory, the advantage of AIMD simulations for the present purpose is their ability to accurately capture mutual solute–solvent polarization effects, crucial for the adequate reproduction of the details of experimental IR spectra.

**Figure 4:**
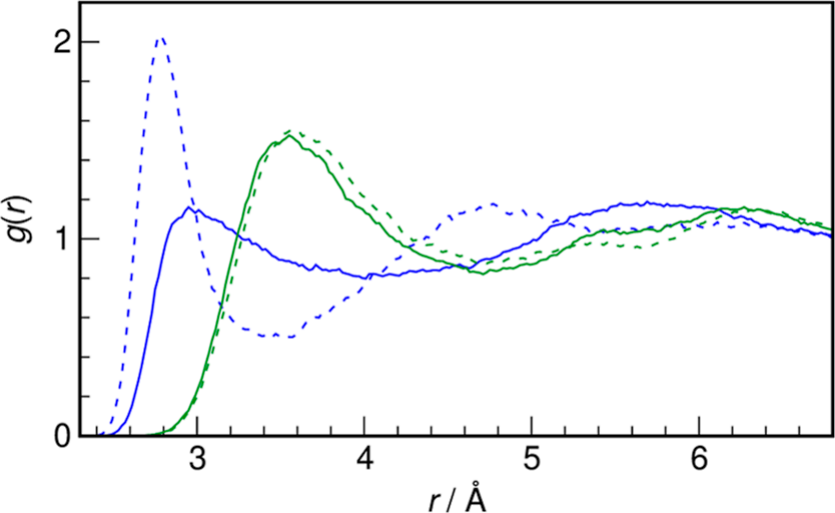
Radial distribution functions for the O···O_w_ pairs (blue) and C···O_w_ pairs (green) for MSM (solid lines) and DMSO (dashed lines, ref (20)), obtained from AIMD simulations.

The structure of the hydration shells of MSM obtained from AIMD simulations is shown in Figure 4 in the form of radial distribution functions (RDFs). The first hydration shell around the O atom of the solute is extremely weak, with on average 5.6 water atoms (O_w_) located within the cutoff at the first minimum in RDF (4.01 Å). The first maximum in the RDF, located at 2.95 Å, hints at very weak solute–water hydrogen bonds. The hydrophobic hydration shell around the C atom of MSM contains 11 O_w_ atoms within the first hydration shell (cutoff 4.71 Å). The most probable C···O_w_ distance is 3.55 Å.

**Figure 5:**
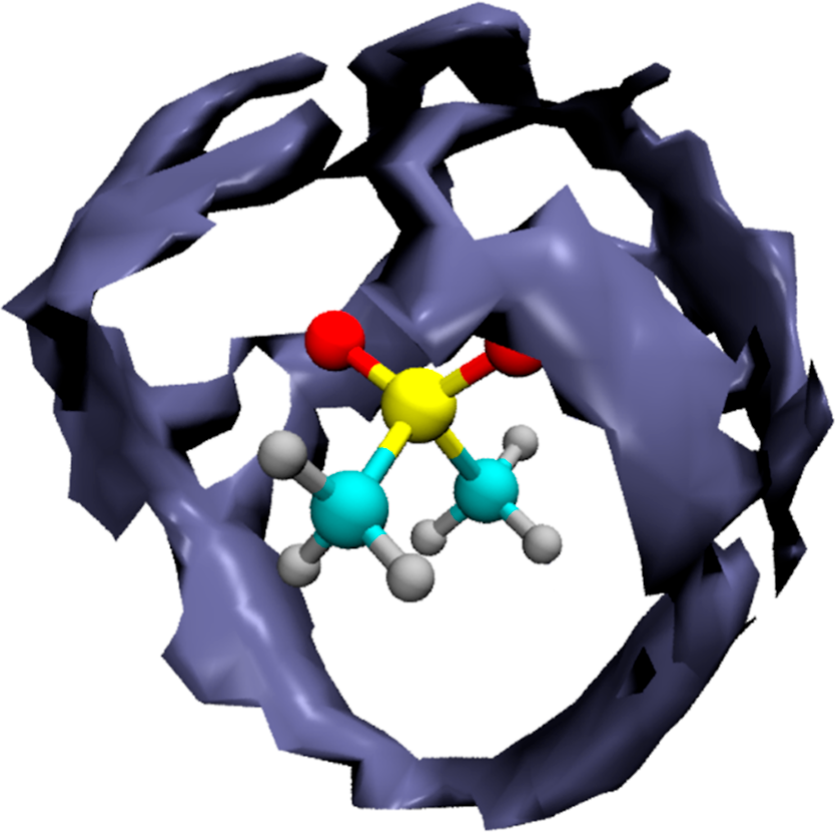
Spatial distribution function of water oxygen atoms around MSM obtained from AIMD simulations. The reference frame is defined by the central molecule and the surface is plotted for *g*(*r*) = 2.3.

The spatial location of the water O atoms in the reference frame of the MSM molecule is shown as a spatial distribution function (SDF) in Figure 5. The plot has been obtained with an isosurface set at 2.3 times the bulk water density. The hydration structure around MSM O atoms is visible as well-defined increased density rings above and around each MSM oxygen. Most interestingly, however, there is a well-defined connecting structure between the two rings, located roughly perpendicular to the C–S–C plane. The two hydration rings around the MSM O atoms are thus connected with a linking water chain that also clearly belongs to the intersection of the hydrophobic hydration shells of methyl groups of MSM.

**Figure 6:**
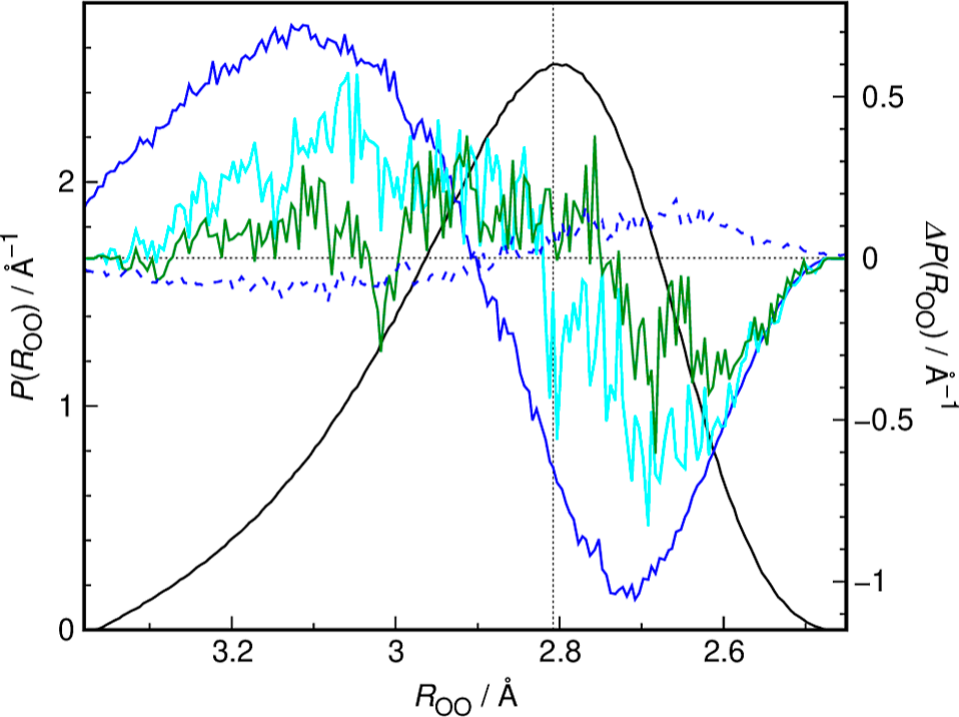
Interatomic oxygen–oxygen distance distribution function obtained from AIMD simulations for hydrogen bonded water molecules in bulk D_2_O (black, left ordinate axis), *P*(*R*_OO_), together with the distance distribution differences with respect to bulk D_2_O (right ordinate axis), Δ*P*(*R*_OO_), for: O···O_w_ hydrogen bonds in MSM (blue solid line), O···O_w_ hydrogen bonds in DMSO (blue dashed line, ref (20)), O_w_···O_w_ hydrogen bonds formed by water hydrogen bonded to the MSM oxygen (cyan), and O_w_···O_w_ hydrogen bonds in the hydration shells of methyl groups of MSM (up to 4.7 Å, green). The cyan and green curves are scaled by a factor of 10 to facilitate easier comparison. The vertical dotted line corresponds to the most probable oxygen–oxygen distance in bulk D_2_O (*R*_OO_ ≈ 2.81 Å).

The extent to which MSM disrupts the hydrogen bonded network of water may be fully appreciated from Figure 6. It is conceptually similar to Figure 3 based on experimental spectra and illustrates the differences between the probability distributions of oxygen–oxygen distance for hydrogen-bonded water molecules in different environments. Here, we used the hydrogen bond definition based on potential energy surface^64^ and adapted from our previous work on DMSO hydration.^20^ As clearly seen, the hydrogen bonds between MSM oxygen and water are extremely elongated, with the population of very weak hydrogen bonds (*R*_OO_ > 2.9 Å) substantially increased and a concomitant decrease of the population of hydrogen bonds of average and below average length. The distance distribution difference is qualitatively very similar to the one based on measured FTIR spectra; cf. Figure 3b. This shift of the hydrogen bond distance distribution toward higher *R*_OO_ values for water molecules directly bonded to MSM oxygen atoms also has lasting consequences for further hydration shells. As seen in Figure 6, both hydrogen bonds to further water molecules formed by those water molecules that are already bonded to MSM, as well as water–water hydrogen bonds in the hydrophobic hydration shells of the methyl groups of the solute display qualitatively the same pattern of distance distribution difference with respect to bulk water. Therefore, the weakening of the immediate hydration shell of MSM oxygen atoms is pronounced enough to propagate to further water molecules, effectively disrupting also the hydrophobic hydration shell that would normally form a clathrate-like structure.

While obtaining the IR spectrum of the system from AIMD simulations is a routine task when dipole moments are available,^60^ we are mostly interested in comparison of the simulated IR spectra to the experimentally obtained spectrum of MSM-affected water. This is made possible by considering the dipole moment of a spherical cluster centered on the solute, with the dipole moments of water molecules added using a smooth cutoff function, allowing for fractional counting. The result is the distance-dependent IR spectrum of the solute–water cluster at the selected cutoff radius *R*_c_, see refs (61 and 62) for details of the procedure. Such ε_R_(ν, *R*_c_) spectra provide a smooth transition from the solute IR spectrum (at *R*_c_ → 0) to the one of a bulk system (at *R*_c_ → ∞). However, the most interesting is the intermediate region, where by increasing *R*_c_ we are able to probe the growing hydration shell.^61^

**Figure 7:**
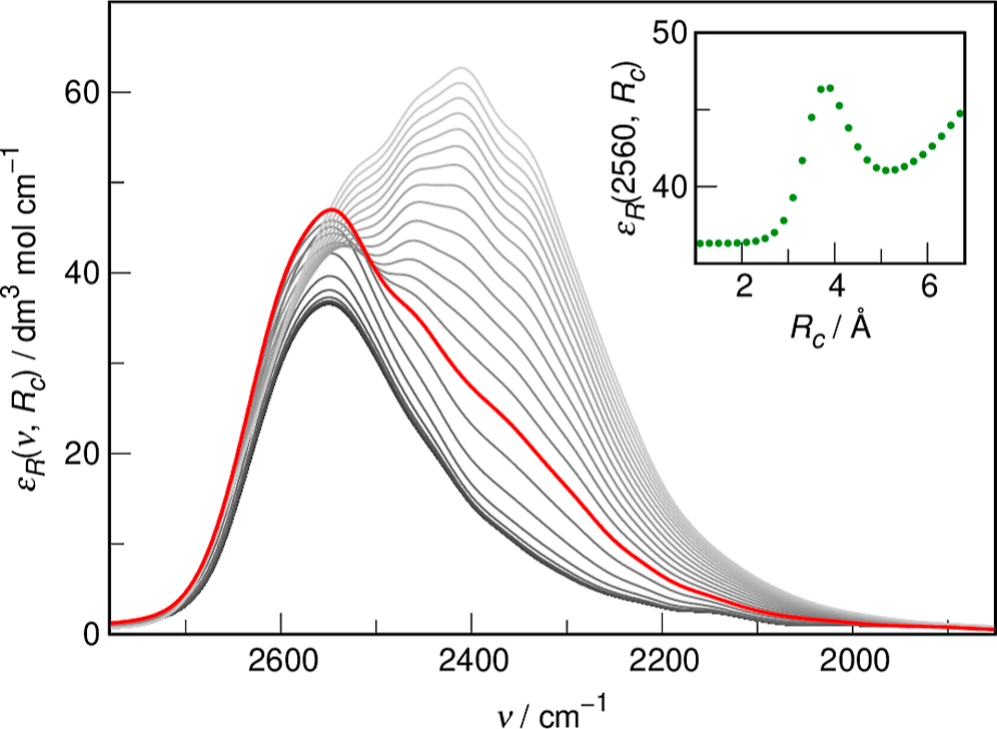
Distance-dependent IR spectra from AIMD simulations at the cutoff radii *R*_*c*_ ranging from 0.1 Å up to 6.7 Å at every 0.2 Å. Lighter shades of gray indicate increasing *R*_c_ values. The ε_R_(ν, *R*_c_ = 3.9 Å) spectrum indicated in red. The inset shows the dependence of the intensity of the distance-dependent IR spectrum on *R*_c_ at the probing wavenumber ν° = 2560 cm^–1^ (the position of the maximum at *R*_c_ → 0).

The obtained distance-dependent IR spectra are shown in Figure 7. It is readily apparent that the ε_R_(ν, *R*_c_) spectrum changes continuously from the spectrum of an “isolated” solute without any water at *R*_c_ ≈ 0 to a bulk-like spectrum at *R*_c_ → *L*/2, which is the actual cutoff limit in a cubic simulation cell. Note that the spectrum of the solute actually shows water bands due to the mutual polarization effects in the system. This fact is noticeable even for monatomic ions that lack their own intramolecular vibrations.^61,62^

The ε_R_(ν, *R*_c_ = 0.1 Å) shows a single maximum at ν° = 2560 cm^–1^, significantly blue-shifted from the bulk D_2_O spectrum obtained by us from AIMD simulations (ν° = 2417 cm^–1^).^20^ Note that the terms “red shift” and “blue shift” as used here and below always refer to the position at maximum of the ν_OD_ band in the computational IR spectrum of liquid D_2_O. Notably, the molar absorption coefficient at 2560 cm^–1^ is heavily modulated by the hydration shell of MSM (see inset in Figure 7). The cutoff radius, for which ε_R_ reaches a local maximum at 2560 cm^–1^, i.e. *R*_c_° = 3.9 Å, can be interpreted as the size of the cluster that is maximally affected by the central solute and thus its spectrum (shown in red in Figure 7) serves as a computational analogue of the MSM-affected water spectrum determined from experimental data. The average fractional number of water molecules contained within *R*_c_° is *N*° = 3.3 ± 0.7, in perfect agreement with the affected number obtained from FTIR spectra, cf. Table 1. While the shift of the maximum of the band from bulk water is much greater in the distance-dependent spectrum at *R*_c_° than in experiment (143 and 87 cm^–1^, respectively), we found previously that AIMD simulations not accounting for nuclear quantum effects systematically overestimate the band shifts by a factor of 1.8.^62^ Taking this into account, the corrected band shift in the distance-dependent spectrum is 79 cm^–1^, in very good agreement with the experimental one.

**Figure 8:**
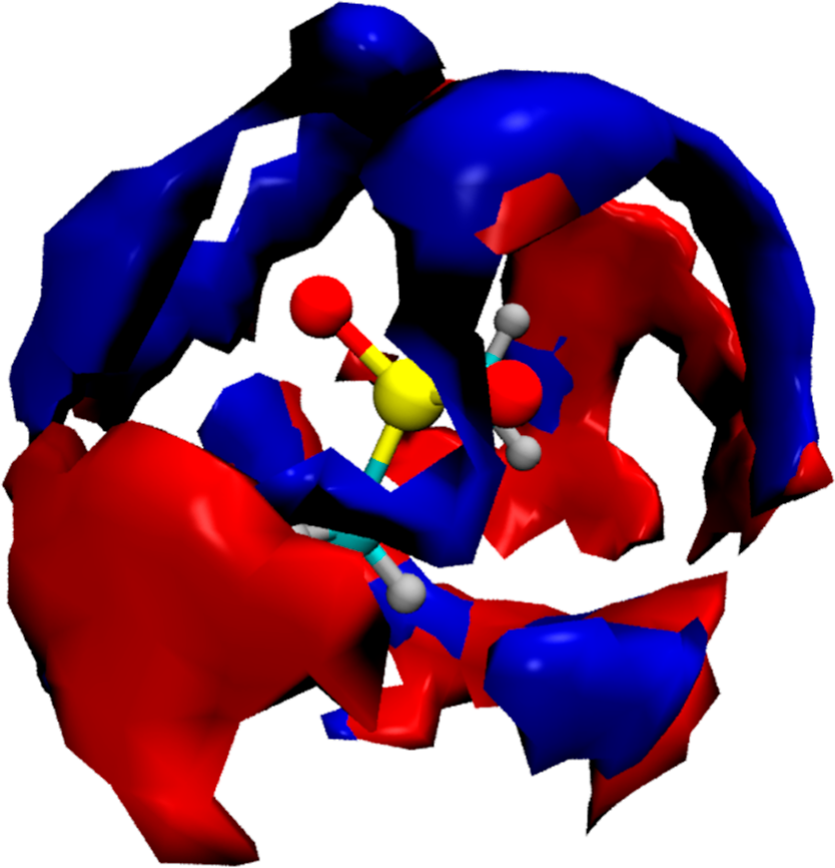
Spatially resolved IR autocorrelation spectrum of D_2_O around MSM at ν = 2360 cm^–1^ (red) and ν = 2560 cm^–1^ (blue) obtained from AIMD simulations. The reference frame is defined by the central molecule, and the intramolecular contribution of the solute at the origin is removed for clarity.

Another possibility of the spatial analysis of IR spectra in solute–solvent systems is provided by spatially resolved IR autocorrelation spectrum based on molecular dipole density smoothly distributed on a Cartesian grid.^62,65^ This spectrum captures local IR excitations in the reference frame of the solute, thus enabling the visualization of particularly strong IR intensities confined to specific spatial regions. The spatially resolved IR autocorrelation spectrum of the studied system is shown in Figure 8.

The red and blue surfaces show the IR intensity at 2360 and 2560 cm^–1^, respectively, i.e., at positions red- and blue-shifted from the bulk water spectrum. The cutoff intensity values were chosen to be the same for both surfaces. There exists a striking correspondence between the blue surface, depicting the spatial regions with the strongest IR intensity at 2560 cm^–1^, and the SDF of water molecules around MSM (see Figure 5). Consequently, the regions where the probability of finding a water molecule is the highest are also regions where the IR intensity at the band position maximum of MSM-affected water is the strongest. This readily explains why the affected water spectrum is dominated by the strongly blue-shifted component band, both in the experimental and distance-dependent IR spectra, cf. Figures 1 and 7. What is more, the spatially resolved IR autocorrelation spectrum also helps with visualizing regions, where a particularly strong IR intensity may be expected for the red-shifted component of the MSM-affected spectrum. The red surfaces effectively link the rings of the water molecules solvating the MSM oxygen atoms, as seen in Figure 8. Comparison with SDF reveals that the local water density is much smaller there, thus explaining the weak intensity of the red-shifted component band in the experimental affected spectrum; cf. Figure 1. This is also the expected location of the water link between the two oxygen atoms of MSM that shows shorter *R*_OO_ distances, and consequently red-shifted band positions, identified in the optimized cluster structures, as seen in Figure 2. Thus, we obtain complementary evidence from MSM-affected water spectrum, optimized cluster structures, and spatially resolved IR spectrum proving the existence of shorter, stronger hydrogen bonds responsible for the red-shifted component band in the affected spectrum even for such weakly hydrated solute as MSM.

### Estimation of MSM Gutmann’s Donor Number

The DN proposed by Gutmann^66^ allows us to characterize the electron donor properties of chemical substances. It is defined as the dimensionless negative value of the enthalpy change in kcal·mol^–1^ for the 1:1 adduct formation of the electron donor solvent (S) with antimony (V) pentachloride (SbCl_5_), as the electron acceptor, in a highly diluted solution in 1,2-dichloroethane: DN = −Δ*H*(S·SbCl_5_). DN value measures the Lewis basicity of the solute and also correlates linearly with the Kamlet and Taft’s β solvatochromic parameter, measuring the hydrogen bond accepting ability of a molecule.^67^ Thus, it offers a convenient way of comparing the strength of hydrogen bond acceptors in an aqueous environment.

The DN calculation method returns DN values fitted to various models. As in the original publication,^44^ the model with explicit solvation and perturbed chemical hardness gives the lowest root-mean-square deviation for the fit of the literature data. Raw output values of DNs for DMSO and MSM are, respectively, 30.0 (which is slightly larger than the experimental value of 29.8) and 17.7. Due to structural similarity of DMSO to MSM, it was assumed that a similar overestimation of DN takes place. The difference between fit and reference values of DMSO’s DN was used to apply correction to the DN of MSM, resulting in the final value of 17.5 ± 3.6.

## Discussion

### MSM Hydration vs DMSO Hydration

Although, in terms of chemical structure, MSM differs from the DMSO molecule in the presence of an additional oxygen atom attached to a sulfur atom, there are clear differences in the hydration of these molecules. The influence of these solutes on the structure of water categorized them into two different groups: MSM is a “structure-breaking” solute, while DMSO belongs to the “structure-making” solutes.

As seen from Figure 4, the major differences in first hydration spheres of both solutes come primarily from the hydration shell around the solute’s oxygen atom(s), as seen in the *g*(*r*) curve for the O···O_w_ pairs. We found previously for aqueous DMSO that the first hydration shell of the solute’s oxygen is well-defined, with a prominent sharp maximum at 2.77 Å (i.e., less than the O_w_···O_w_ distance in bulk water) and a coordination number of 3.5.^20^ In striking contrast, the respective RDF for MSM reveals a poorly defined, broad first peak with a maximum shifted to 2.95 Å, revealing much weaker interactions between its oxygen atoms and water. This corresponds with the number of hydrogen bonds formed between solute oxygen atom and water which falls from 2.5 for DMSO^20^ to 1.2 (per oxygen) for MSM. On the other hand, the differences in the hydrophobic hydration shell around the methyl groups of both solutes are relatively minor, see Figure 4. Nevertheless, a slight decrease of the coordination number is observed, from 11.6 for DMSO to 11.0 for MSM. This is accompanied by a decreased population of stronger hydrogen bonds in the hydrophobic hydration shell in the latter case, cf. Figure 5.

Moreover, the hydrogen bond distribution around both solutes is different, as illustrated by the differences in the distribution of oxygen–oxygen distances, Δ*P*(*R*_OO_), between water affected by these solutes and bulk water (Figure 3b). In the case of MSM, the differences in oxygen–oxygen distances relative to pure water are much greater than those obtained for DMSO,^20^ which means that MSM influences the structure of water in its surroundings to a greater extent. The analysis of the Δ*P*(*R*_OO_) indicates that in the presence of MSM, the population of strong water hydrogen bonds (oxygen–oxygen distances of ca. 2.75 Å) slightly decreases in comparison to bulk water. In addition, the population of water–water hydrogen bonds with the most likely distance in pure water (ca. 2.83 Å) is significantly reduced. At the same time, a significant increase in the population of water molecules with longer oxygen–oxygen distances (ca. 3.0 Å) can be seen, which is consistent with the conclusion that hydrogen bonds are weakened in the MSM hydration sphere. This difference in hydrogen bond populations of various lengths is also fully corroborated by results of AIMD simulations, where the same pattern of changes in oxygen–oxygen distance distribution for hydrogen bonded water molecules was found; see Figure 6. As seen in this figure, these changes are also repeated for hydrogen bonds in different situations, specifically those between a water molecule hydrogen bonded to MSM oxygen and other water molecules, as well as water–water hydrogen bonds in the hydrophobic hydration shell.

In turn, in the DMSO hydration sphere, an increased population of strong hydrogen bonds can be observed compared to pure water, i.e., enhancement of water hydrogen bonds (Figure 3b). The presence of this population is the result of the cooperation of hydrogen bonds between water molecules involved in creating a cage around the methyl groups with water molecules interacting with the sulfonic oxygen atom.^20^ We also noticed a similar effect in the case of *N*-methylacetamide (NMA).^68^ Based on previous works,^20,68^ we found that the condition for the existence of this population is the same strong interaction of water molecules with the hydrophilic group as between water molecules in the ice structure. The strong interaction of water molecules with the sulfoxide group of DMSO “anchors” a common network of hydrogen bonds on this group, ensuring stabilization of the strengthened DMSO hydration sphere.

The absence of the discussed enhancement in the case of MSM (Figure 3b) indicates anticooperativity of hydrogen bonds of water molecules interacting with the sulfone group and water molecules around the methyl groups of MSM. Water molecules involved in direct interactions with MSM oxygen atoms form very weak hydrogen bonds. The lack of “anchoring” of the hydrogen bond network on the sulfone group of MSM causes the MSM hydration sphere to be unstable. Water molecules are more likely to form hydrogen bonds with each other than with the solute, as a result of which MSM is poorly hydrated. Nevertheless, there is still a small population of water molecules that give rise to the red-shifted component band of the MSM-affected HDO spectrum, as seen in Figure 1. As inferred from complementary results of DFT cluster optimizations and spatially resolved IR spectra (cf. Figures 2 and 8), they can be pinpointed to a specific spatial region around MSM and attributed to the water chain linking the MSM oxygen atoms first hydration spheres.

### Hydration of Organic Solutes with Different Donor Properties

The DN can provide information about the ability of the solute to interact with surrounding water molecules. In the gaseous state, a water molecule’s DN value is 18.^66^ However, it can be deduced that this value increases to 26.7 in an aqueous solution^69^ owing to the cooperativity of water hydrogen bonds. The donor centers of the solute molecules need to have a DN value of a minimum of 26.7 to effectively hold the clathrate-like water layer around the neighboring nonpolar groups. The DN for MSM (17.5) is much lower than the DN value for liquid water, indicating the lack of “anchoring” of the hydrogen bond network on the sulfone group of MSM. The paragraph below elaborates on this conclusion.

The clathrate-like hydration of small molecules of nonpolar solutes in water seems to have a similar energy state of water’s hydrogen bonds as in the ice structure.^12^ However, completely nonpolar substances are very poorly soluble in water due to the high entropic cost of forming such highly organized water structures. Solubility is significantly improved by the presence of a hydrophilic group in the solute including an electron pair donor. The interaction of water with such a center introduces disorder in the organization of the hydration sphere, which affects the solubility of the solute. It also specifies the strength of the sphere’s structure, as measured by the average energy or length of hydrogen bonds. The stability of this hydration sphere is influenced by the possibility of organizing water molecules in a clathrate-like fashion around the nonpolar groups of the solute. The direct proximity of the electron-donor group to the nonpolar group facilitates the formation of a hydrophobic-type network.^20,68^ This behavior of hydration water molecules is consistent with the concept of “anchored clathrate water” mechanism formulated by Garnham et al.^70^ and recently confirmed by Zielkiewicz.^71^ However, the hydrophilic group must interact with the water molecule at least as strongly as in the ice structure, which also corresponds to the energy of the water–water interaction in the cage around the nonpolar group. It is this hydrogen bond that determines the proper anchoring of the entire network and is the center of interaction by means of which the ice-like structure expands around the neighboring nonpolar groups. In the case of a weaker interaction of a water molecule with a hydrophilic group, as in the case of MSM, there will be a lack of such a core for the expansion of the ice-like hydrogen bond network around the solute. As a result, a water “structure breaking” effect is observed. When the interaction of water molecules with the hydrophilic group is stronger than in the case of ice, then a general strengthening of the hydrogen bonding network of hydration water relative to the situation in the ice structure is observed. Such an effect is consistent with the phenomenon of cooperativity of hydrogen bonds and occurs, for example, in the hydration spheres of NMA,^68^*N,N,N*-trimethylglycine (betaine),^72^ and trimethylamine *N*-oxide.^73^

## Conclusions

In this study, we characterized the hydration shell of methylsulfonylmethane (MSM) by means of FTIR spectroscopy supported by theoretical methods: DFT calculations and AIMD simulations. The results of theoretical calculations helped in the interpretation of spectral results and provided additional information about the hydrogen bonding network of water molecules around the MSM. The agreement between experimental and computational results was satisfactory and provided a consistent picture of MSM hydration in an aqueous solution.

Two populations of water molecules can be distinguished in the hydration sphere of MSM: the first corresponds to water molecules that form very weak hydrogen bonds with MSM oxygen atoms, and the second is related to water molecules that form strong hydrogen bonds with each other around the hydrophobic groups of MSM. However, in general, the structure of water in the immediate vicinity of MSM is weakened compared to pure water due to the dominant share of very weak hydrogen bonds. This property allows MSM to be categorized as a water “structure breaking” solute. Furthermore, in comparison to pure water, we noticed a decrease in the population of strong hydrogen bonds between water molecules around the methyl groups of the solute. The formation of the hydrophobic hydration sphere and, consequently, the entire water hydrogen bond network surrounding the MSM is determined by the interaction of water molecules with the hydrophilic sulfone group of the MSM. Extremely weak hydrogen bonds formed by water molecules with MSM oxygen atoms hinder the formation of an “ice-like” hydration sphere. In such a situation, the hydrophilic group is unable to maintain a common network of water–water hydrogen bonds. As a result, the MSM hydration sphere is unstable.
